# Conservative Treatment of Pediatric Fractures—Narrative Review of Acceptable Deformity and Remodeling Potential

**DOI:** 10.3390/medsci14040432

**Published:** 2026-07-25

**Authors:** Marko Bašković, Jana Buzuk, Bianka Dujić, Danijela Jurić, Kristina Jurković, Karla Pehar, Sara Vuković

**Affiliations:** 1 School of Medicine, Catholic University of Croatia, Ilica 242, 10000 Zagreb, Croatia; 2 Department of Pediatric Surgery, Children’s Hospital Zagreb, Ulica Vjekoslava Klaića 16, 10000 Zagreb, Croatia; 3 School of Medicine, University of Zagreb, Šalata 3, 10000 Zagreb, Croatia; 4 Scientific Centre of Excellence for Reproductive and Regenerative Medicine, School of Medicine, University of Zagreb, Šalata 3, 10000 Zagreb, Croatia; 5 Croatian Academy of Medical Sciences, Kaptol 15, 10000 Zagreb, Croatia; 6 Referral Center for Pediatric Traumatology, Children’s Hospital Zagreb, Ulica Vjekoslava Klaića 16, 10000 Zagreb, Croatia

**Keywords:** pediatric fractures, conservative treatment, bone remodeling, physis, growth plate, acceptable angulation, nonoperative management, children

## Abstract

Fractures are among the most common injuries in childhood, yet the immature skeleton differs fundamentally from the adult skeleton. A thick periosteum, open physes, and a powerful capacity for remodeling mean that most pediatric fractures unite and realign without surgery. Despite this, national registries document an increasing use of operative fixation, including in some fracture groups with substantial remodeling potential. This narrative review synthesizes the contemporary evidence on the limits within which fractures throughout the growing skeleton can be treated conservatively. The literature was identified through structured searches of PubMed, Scopus, Web of Science, and the Cochrane Library. For each region we summarize the accepted thresholds of angulation, rotation, shortening, and displacement, together with the age- and site-specific modifiers that determine whether a deformity will remodel, and we present these thresholds in comparative tables. We also delineate the genuine indications for surgical treatment, so that the boundary between conservative and surgical management is defined rather than blurred. A secure command of these region-specific limits lets the treating physician exploit the child’s remarkable remodeling potential and avoid both under- and over-treatment.

## 1. Introduction

Fractures are among the most frequent injuries of childhood, and several national registries have documented a steady rise in their incidence over recent decades [1,2]. The immature skeleton, however, is not simply a scaled-down version of the adult skeleton. A thick and biologically active periosteum, one or more open physes that drive longitudinal growth, and a powerful capacity for remodeling together separate pediatric from adult trauma and render precise anatomical reduction far less decisive for the final result than it is in adults [3,4]. These same properties underpin a principle that generations of surgeons regarded as self-evident, that the great majority of fractures in children can be treated without an operation, achieving a functional and cosmetic outcome that equals or surpasses that of internal fixation, while sparing the child the hazards of anesthesia and surgery [4,5]. The distal radius is the single most commonly fractured bone of childhood, accounting for roughly 20–30% of all pediatric fractures, with a peak at the pubertal growth spurt [6,7,8]. Fractures affect a large minority of all children, boys more often than girls, and their site shifts predictably with age, from the clavicle and tibia of infancy to the distal radius of the school years [9,10].

In recent years, however, the pendulum has swung conspicuously toward surgery. Analyses of national and institutional databases show a marked increase in the operative treatment of children’s fractures, most prominently for diaphyseal forearm fractures, and in the ambulatory setting the use of operative fracture care in children roughly tripled over a single decade [2,11]. The reasons are multifactorial, spanning technological, economic, social, and medico-legal factors and, not least, the expectation of a ‘perfect’ radiograph, and evidence-based medicine is by no means the dominant driver [5]. At the same time, part of this rise probably reflects legitimate developments rather than overtreatment alone, including better recognition of unstable injuries, improved anesthetic and fixation techniques, the treatment of older and heavier adolescents, higher patient and family expectations of an early return to activity, and wider access to specialized pediatric trauma care. Surveys reveal wide disagreement among pediatric orthopedic surgeons, not only over the choice between operative and nonoperative treatment but even over whether and when to attempt closed reduction [5,12].

The consequences of this shift are not trivial. Every operative procedure or closed manipulation of a child’s fracture requires general anesthesia; carries the risks of infection, delayed union, implant-related complications, and a second procedure for implant removal; and is substantially more expensive than a cast [5,12]. Several series report complication rates rising in parallel with operative rates [2,12]. Set against this is the single most important biological fact of the growing skeleton, that its remodeling capacity is enormous, yet this potential is increasingly disregarded, and there is concern that some children undergo operative treatment for deformities that might have remodeled spontaneously, although direct high-level evidence quantifying this across regions remains limited [3,4].

This narrative review aims to gather the contemporary evidence on the limits within which pediatric fractures may be managed conservatively. For each anatomical region we summarize the accepted thresholds of angulation in the anteroposterior and lateral planes, of rotation, shortening, and displacement, together with the age- and sex-dependent modifiers that decide whether a given deformity will remodel. Our intention is not to discourage surgery where it is genuinely indicated, but to give the treating physician a clear, evidence-based map of what can safely be entrusted to the remodeling power of the child’s own skeleton. Framed as a single question, within what region-, age-, and plane-specific limits can a pediatric fracture safely be entrusted to conservative care, this review is intended for the clinicians who make that decision at first contact, including pediatric and general orthopedic surgeons, emergency and pediatric physicians, and trainees. Its practical contribution is a consolidated, region-by-region reference of acceptable deformity thresholds set beside the genuine indications for surgery, so that the boundary between conservative and operative care can be applied consistently at the bedside.

### Search Strategy and Evidence Appraisal

This article is a narrative review and does not follow the protocol-driven methodology of a systematic review. Its purpose is to synthesize and contextualize a broad, clinically oriented literature rather than to answer a single quantitative question. To make the process transparent, the electronic databases PubMed/MEDLINE, Scopus, Web of Science, and the Cochrane Library were searched from inception to June 2026. The search combined controlled-vocabulary and free-text terms for the pediatric population (“child”, “children”, “pediatric”, “adolescent”, “immature skeleton”) with terms for fracture management and remodeling (“fracture”, “conservative treatment”, “nonoperative”, “closed reduction”, “cast”, “remodeling”, “acceptable angulation”, “malunion”, “growth plate”, “physis”), together with anatomical qualifiers for each region. The reference lists of retrieved articles and of standard textbooks were hand-searched to identify further sources. Only publications in English were included. Where sources reported discrepant thresholds for the same fracture, the range is reported, and, where one exists, the more recent or higher-quality source is indicated.

The strength of the evidence behind the recommendations that follow is uneven, and this should be kept in mind whenever any single figure is applied. Some rest on robust contemporary evidence from randomized trials and systematic reviews. Others derive largely from long-standing clinical practice, from biomechanical reasoning, or from older series rather than from comparative studies and are better regarded as pragmatic guidance than as validated cut-offs. Throughout this text we have therefore tried to indicate in words where a threshold is well supported and where it is not.

The numerical thresholds presented throughout this review are clinical guides rather than rigid cut-offs, and each must be applied in the light of the individual child’s remaining growth, skeletal maturity, fracture stability, plane of deformity, and functional demands, as set out in Section 2.2. The breadth of anatomical coverage adopted here, from the craniofacial skeleton to the foot, is a deliberate strength for a single clinical reference, but it inevitably limits the depth achievable for any one region.

## 2. Biological Basis of Conservative Treatment

### 2.1. Periosteum, Physis, and the Phases of Healing

Three features of the growing skeleton account for the forgiving behavior of pediatric fractures. These are a thick periosteum, one or more open physes, and a vigorous remodeling response [3,13]. The child’s periosteum is thick, strongly osteogenic and only loosely attached to the diaphysis, while adhering densely at the physeal margin. Because it seldom ruptures around its whole circumference, an intact periosteal hinge usually persists on the concave side of the injury. This hinge limits initial displacement, stabilizes the fragment during and after closed reduction, and provides a scaffold for the rapid callus that is characteristic of children. Occasionally the same sleeve, when interposed in the fracture gap, becomes an obstacle to reduction [3]. Fracture healing then proceeds through three overlapping phases of inflammation, repair, and remodeling, the last of which is far more prolonged and powerful in the child than in the adult [3,13].

### 2.2. Determinants and Limits of Remodeling

Remodeling of a malunited fracture is achieved by two mechanisms working together. Reorientation of the physis accounts for approximately three-quarters of angular correction, and appositional ‘cortical drift’ along the diaphysis in accordance with Wolff’s law contributes the remaining quarter. This roughly three-to-one split is an approximation drawn from clinical and experimental observation, and the actual contribution of physeal reorientation varies with the site of the fracture, the proximity and activity of the physis, and the plane of the deformity rather than representing a fixed universal ratio [3,13]. This capacity is neither universal nor unlimited, and six determinants govern how much deformity may safely be entrusted to spontaneous correction.

- Skeletal (physiological) age and remaining growth: the younger the child and the more growth remaining, a working rule is at least two years, the greater the correction.
- Activity of the nearest physis: a fracture close to a highly active growth plate (e.g., the distal radius) remodels well, whichever limb it lies in; one remote from any physis remodels poorly.
- Growth contribution of that physis: physes responsible for a large share of a bone’s length, such as the proximal humerus and distal radius, tolerate more deformity.
- Plane of the deformity: angulation in the plane of the adjacent joint’s motion corrects reliably, whereas coronal-plane angulation corrects less well.
- Character of the deformity: rotational malalignment and intra-articular incongruity remodel poorly and unpredictably and should generally not be accepted.
- Body habitus: a high body-mass index makes reduction harder to achieve and hold and is an independent risk factor for loss of reduction, so heavier children warrant closer follow-up.

First, skeletal age matters, and the younger the child, the greater the potential, so as a working rule at least two years of growth should remain for meaningful correction. Because it is remaining growth rather than chronological age that drives correction, skeletal maturity should be judged directly rather than inferred from age alone. Second, the activity of the nearest physis matters more than any simple proximal-to-distal rule, and a fracture close to a highly active growth plate remodels well even in the distal limb, with the distal radius being the classic example of a distal site with excellent remodeling capacity, whereas a fracture remote from any physis remodels poorly wherever it lies. Third, the growth contribution of that physis matters, and a physis responsible for a large share of a bone’s length, such as the proximal humerus or the distal radius, supports more correction than one that contributes little to longitudinal growth [3,13]. Fourth, the plane of the deformity matters, and angulation lying in the plane of movement of the neighboring joint corrects reliably, whereas coronal-plane angulation corrects less well. Fifth, the character of the deformity matters, and rotational malalignment and intra-articular incongruity remodel poorly and unpredictably and should generally not be accepted. Differences between the upper and lower limb in this respect are better explained by the activity of the physis nearest the fracture and by biomechanical loading than by any blanket rule that one limb remodels better than the other [13]. Quantitatively, angular malunions of the distal radius remodel at a mean rate of roughly 1–2.5° per month along an exponential curve whose initial velocity is greater the larger the deformity, so that even malunions beyond the conventional 15° limit correct substantially over one to two years [14,15,16]. Animal and clinical studies nonetheless confirm that this realignment is only partly reliable, correcting angular deformity in the plane of joint motion but neither rotational malalignment nor deformity outside that plane, so remodeling must be anticipated rather than assumed [17]. Sixth, body habitus matters. A high body-mass index makes a closed reduction harder to achieve and to hold in a cast and is an independent risk factor for loss of reduction across several fracture types, so heavier children warrant closer follow-up and a lower threshold for fixation [18]. Finally, a satisfactory clinical result may arise through three mechanisms that should not be conflated. The first is true osseous remodeling, which restores bony alignment. The second is functional compensation by adjacent joints, conspicuous at the mobile shoulder, where considerable residual deformity of the clavicle or proximal humerus is well tolerated. The third is cosmetic adaptation, as the soft-tissue envelope masks a persisting bony prominence. Only the first represents anatomical correction, and the distinction matters when counseling families and interpreting follow-up radiographs. A note on terminology follows from this. Throughout this review ‘infant’ or ‘toddler’ denotes roughly 0–3 years, ‘young child’ about 3–6 years, ‘school-aged child’ about 6–10 years, ‘adolescent’ about 10 years to skeletal maturity, and ‘near skeletal maturity’ the final one to two years of growth. Because it is remaining growth rather than chronological age that governs remodeling, these bands are deliberately approximate, and body weight is stated wherever it materially influences the choice of treatment.

### 2.3. Physeal Injuries and the Salter–Harris Classification

Between 15% and 20% of all pediatric fractures involve a physis [19]. Biomechanically, the physis is the weakest link of the immature skeleton, being most resistant to compression and least resistant to shear and torsion, so it tends to fail during the adolescent growth spurt when it is at its most vulnerable. The classification described by Salter and Harris in 1963 remains the everyday working framework. Types I and II, which together form the large majority and spare the germinal layer, carry a low risk of growth arrest and can usually be managed by gentle closed reduction and immobilization. Types III and IV are intra-articular and, together with the type V crush injury, carry the highest risk of premature physeal closure and generally require anatomical, often operative, reduction [19,20]. A modest residual angulation after a physeal fracture may itself remodel, but this must be weighed against the risk of a physeal bar. The child should therefore be followed until symmetrical Park–Harris growth-arrest lines confirm that growth has resumed evenly [13,19].

## 3. General Principles of Conservative Management

### 3.1. Assessment and the Concept of “Acceptable” Deformity

The decision to treat a fracture without operation rests on a single question. Will the expected final deformity lie within limits that the child’s remaining growth will correct, or that will not impair function or appearance? “Acceptable” alignment is therefore not a fixed number but a range that depends on the bone, the age of the child, and the plane of the deformity, as set out above [3,13]. The thresholds compiled in this review are guidelines rather than rules. Remaining growth, functional demands, and the family’s expectations must all be weighed, and a limit appropriate for a four-year-old is rarely appropriate for a fourteen-year-old [3,12]. Rotational malalignment, intra-articular step-off, and deformity outside the plane of joint motion are the least forgiving and should lower the threshold for intervention [13]. Crucially, acceptable frontal- and sagittal-plane alignment on radiographs does not by itself imply acceptable rotation. Rotational deformity is easily overlooked on standard views, remodels poorly and unpredictably, and must be judged clinically, most importantly in the forearm and hand, where even modest malrotation produces disproportionate functional loss [13,17].

### 3.2. Closed Reduction, Casting Technique, and the Cast Index

For the majority of displaced fractures in children, closed reduction and cast immobilization remain the treatment of choice. Reduction maneuvers reverse the mechanism of injury and are performed under adequate analgesia, whether procedural sedation, a regional block or, where necessary, general anesthesia [21]. The intact periosteal hinge is used to stabilize the reduction rather than being disrupted [3]. Maintenance of alignment depends less on the casting material than on the mold. A well-applied cast follows the contour of the limb with thin, even padding and a three-point mold that opposes the deforming force, supplemented by an interosseous mold in the forearm [21]. The quality of the mold can be quantified by the cast index, the ratio of the internal sagittal to the internal coronal diameter of the cast at the level of the fracture, and a value above 0.8 is consistently associated with loss of reduction, so the cast should be molded to an index of about 0.7–0.8 [21,22]. Whether the cast extends above or below the elbow, and for how long, depends on the fracture and the age of the child [21].

### 3.3. Stable Fractures: The Case for Minimal Immobilization

Not every fracture that unites needs a rigid cast. The most common fracture of childhood, the distal radial buckle (torus) fracture, is inherently stable, and several randomized trials and a subsequent meta-analysis have shown that a removable splint, or even a soft bandage with immediate discharge, provides pain relief and functional recovery equivalent to a cast, with greater convenience and no increase in complications [23,24]. The large UK FORCE trial confirmed the equivalence of a soft bandage and rigid immobilization for wrist torus fractures, which heal reliably within three to four weeks [23]. Recognizing which fractures are stable therefore avoids not only surgery but also unnecessary casting, follow-up visits, and repeat radiographs. The strength of this evidence is specific to the distal radial buckle (torus) fracture and should not be extended uncritically to every stable injury. Other intrinsically stable, minimally displaced patterns, such as the toddler’s fracture of the tibia and many isolated metacarpal and phalangeal fractures, may likewise be managed with lighter or removable immobilization, but the supporting evidence is weaker, and the decision should rest on a documented judgment of fracture stability rather than on extrapolation from the torus model [23,24].

### 3.4. Follow-Up, Re-Manipulation, and the Limits of Casting

Displaced fractures held in a cast require radiographic surveillance, typically at one and two weeks, because redisplacement, when it occurs, does so early. A high cast index or an initial complete displacement predicts loss of position, and unacceptable redisplacement then forces a choice between re-manipulation and acceptance in the expectation of remodeling [22]. Repeated manipulation is not benign, and each attempt risks further physeal insult and requires renewed anesthesia, and the evidence increasingly cautions against reflexive re-manipulation of deformities that will remodel [12,13]. Finally, the cast itself is not a risk-free device. Even in experienced hands, casts and splints carry roughly a one-per-cent risk of complications, including pressure sores, thermal and cast-saw injuries, loss of reduction, and, most seriously, compartment syndrome, which in a child may become irreversible within hours. Casting in extreme positions of flexion should be avoided, and pain disproportionate to the injury under a cast must never be dismissed [25]. For physeal injuries specifically, surveillance should continue for six to twelve months, and, for high-risk physes, up to two years, so that a growth disturbance is detected early through the appearance and behavior of a Park–Harris line. Throughout the follow-up, imaging should follow the ALARA principle, obtaining the minimum number of views required to answer a specific clinical question.

### 3.5. When the Fracture Is Not What It Seems: Non-Accidental and Pathological Fractures

Finally, not every fracture in a child is a simple injury, and two situations must be actively excluded before conservative treatment proceeds. The first is inflicted (non-accidental) injury. Certain patterns, such as classic metaphyseal ‘corner’ or ‘bucket-handle’ lesions, posterior rib fractures, and multiple fractures at different stages of healing, carry a high specificity for abuse, and a long-bone fracture in a non-ambulant infant should prompt a skeletal survey and a safeguarding assessment [26,27,28]. The second is the pathological fracture through abnormal bone. A fracture after trivial trauma, or radiographic lucency at the fracture site, should raise suspicion of a unicameral or aneurysmal bone cyst, fibrous dysplasia, osteogenesis imperfecta, or, rarely, malignancy. Benign cystic lesions account for a substantial share of pathological fractures of the proximal humerus and femur and are managed by treating the fracture and then the lesion, whereas a suspected malignant cause must be investigated before any fracture surgery [29,30]. These two situations are considered here not as a digression but because each overrides the ordinary treatment algorithm. An inflicted injury demands child-protection measures alongside fracture care, and a pathological fracture is treated according to its underlying lesion rather than by the acceptable-deformity thresholds that govern routine trauma.

### 3.6. The Late-Presenting Fracture and Established Malunion

A distinct problem is the child who presents late, days or weeks after injury, with a fracture that has already begun to unite in an unacceptable position. The deformity is weighed against the same determinants set out in Section 2.2, but with two added considerations. Early callus can often still be re-manipulated within roughly the first two to three weeks, on occasion with a controlled osteoclasis, whereas a fracture that has united solidly is generally better allowed to remodel and reassessed over the following one to two years than subjected to immediate osteotomy. Whether an established malunion warrants correction turns on the plane of the deformity (angulation in the plane of joint motion is favorable, rotation and intra-articular incongruity are not) and on the growth remaining to drive correction. When correction is genuinely required, it is frequently better deferred until remodeling potential is exhausted, and for suitable peri-articular deformities, guided growth may achieve it without an osteotomy. Families should be counseled that a late presentation does not automatically mandate surgery and that watchful reassessment is often the wiser course [3,5,13].

## 4. Craniofacial Skeleton and Spine

### 4.1. Craniofacial Fractures

Fractures of the facial skeleton illustrate the remodeling principle in its most striking form. In the young child a large cranium-to-face ratio, unerupted tooth germs, and active cartilaginous growth centers make the jaw resilient and facial fractures comparatively uncommon [31]. The mandibular condyle is the paradigm, being simultaneously a fracture site and a growth center with an extraordinary capacity to remodel. Closed functional management, involving a soft diet, a short period of maxillomandibular fixation where required, and functional orthodontic appliances, is therefore the first-line treatment in growing children, whereas open reduction risks injuring the growth center and commonly necessitates later plate removal [31,32]. Imaging series have documented complete condylar remodeling and restoration of ramus height even after fractures displaced by more than 9 mm or angulated by more than 25°, provided the condylar head remains at least partly within the glenoid fossa and functional loading is maintained, and paradoxically, prolonged rigid immobilization may worsen the result [32,33]. These striking figures derive from selected series of extracapsular condylar-neck and low condylar fractures and describe what has been observed to remodel under favorable conditions rather than a general threshold applicable to every condylar pattern. High intracapsular condylar-head fractures, comminuted injuries and fractures with the head displaced out of the glenoid fossa carry a greater risk of growth disturbance, ankylosis, and facial asymmetry and should be presented and interpreted with corresponding caution rather than as a blanket license to accept displacement or angulation of this magnitude [31,32,33]. Anatomical open reduction is reserved for the older adolescent, whose remodeling capacity approaches that of the adult, and for displacement of the condylar head out of the fossa or a fracture that prevents normal occlusion [31,33]. Other facial fractures follow the same conservative logic in the growing child. Nasal fractures are usually treated by closed reduction within the first week, before healing consolidates, and most minimally displaced fractures of the maxilla, zygoma, and orbit are observed, with operative treatment reserved for functional compromise, diplopia from orbital soft-tissue entrapment (the pediatric ‘white-eyed’ blow-out, which requires prompt release), or a malocclusion that will not remodel, because extensive rigid fixation may tether subsequent facial growth.

### 4.2. Spinal Fractures

Spinal injuries account for fewer than 5% of pediatric fractures, and the thoracolumbar junction is the most common site of vertebral-body injury [34]. Greater ligamentous elasticity, more horizontally oriented facet joints, and a canal that is capacious relative to the cord allow the immature spine to tolerate more compression than the adult before neural structures are endangered. The majority of compression fractures and stable burst fractures are accordingly treated conservatively, with analgesia, early mobilization, and a thoracolumbosacral orthosis for approximately six to eight weeks, with good long-term outcomes. Residual anterior wedging is usually modest and partially remodels in the skeletally immature, and prolonged bed rest is contraindicated. Operative stabilization is reserved for unstable patterns, namely disruption of the posterior ligamentous complex, flexion–distraction (Chance) injuries that cannot be held reduced, burst fractures with marked canal retropulsion, and any injury with neurological compromise (Table 1). Precision in these terms matters. A burst fracture is regarded as stable when the posterior ligamentous complex, namely the supraspinous and interspinous ligaments, the ligamentum flavum, and the facet capsules, is intact, when there is neither progressive kyphosis nor loss of vertebral-body height beyond roughly 50%, and when canal compromise is limited. ‘Low’ canal retropulsion is generally taken to mean encroachment of less than about one third of the canal in a neurologically intact patient. Because radiographs and computed tomography image bone rather than ligament, magnetic resonance imaging should be obtained whenever injury to the posterior ligamentous complex is suspected, for instance when there is interspinous widening, facet malalignment, or a flexion–distraction mechanism, since an occult ligamentous disruption converts an apparently stable pattern into an unstable one and alters management. Conservatively managed compression and stable burst fractures should additionally be monitored with upright radiographs during the bracing period to confirm that vertebral-body height and segmental kyphosis are not progressing, since progression signals an occult unstable pattern and warrants cross-sectional imaging and reconsideration of the initial classification [34,35].

## 5. Clavicle, Shoulder Girdle, and Upper Arm

### 5.1. Clavicle

The clavicle is among the most frequently fractured bones of childhood, and the overwhelming majority of injuries, including markedly displaced mid-shaft fractures, unite and remodel with simple symptomatic care [36,37]. Classic and contemporary series document remodeling of even severe deformity, with correction of up to 90° of angulation and 4 cm of overlap reported in displaced mid-shaft fractures in children, and a multicenter study of completely displaced fractures in adolescents aged 10–19 years found that shortening, superior displacement, and angulation improved by 60%, 57%, and 38%, respectively, during follow-up [37,38]. Nonoperative management consists of a sling or figure-of-eight bandage for two to four weeks followed by progressive motion [37]. Meta-analysis and prospective multicenter data show that even for completely displaced, comminuted, or ‘Z-type’ adolescent fractures, surgery confers no durable advantage in patient-reported outcome while carrying higher complication and re-operation rates, largely from implant prominence [36,37]. Operative fixation is therefore reserved for open fractures, neurovascular compromise, and impending skin penetration [37]. The frequently quoted figures of up to about 90° of angulation and 4 cm of overlap derive from selected historical series and describe deformities that have been observed to remodel, not targets to be accepted complacently in routine practice. They apply to the young child with substantial growth remaining and intact neurovascular status, and much of the excellent clinical result reflects functional compensation by the mobile shoulder girdle rather than complete osseous correction (Section 2.2). The contemporary evidence base is nonetheless robust. Systematic reviews and the prospective multicenter FACTS cohort confirm that even completely displaced adolescent mid-shaft fractures fare equally well without surgery on patient-reported outcomes [36,38]. In the younger child in particular, a visible bump or asymmetry from a healing clavicular malunion is a cosmetic rather than a functional problem. It characteristically diminishes as the bone remodels and the soft-tissue envelope matures, and it is masked by functional compensation at the highly mobile shoulder girdle. Cosmetic appearance alone is therefore not an indication for fixation, whose surgical scar, implant prominence, and reoperation rates outweigh any aesthetic gain, and this consideration carries progressively more weight the more growth the child has remaining [36,37,38].

### 5.2. Proximal Humerus

The proximal humeral physis contributes roughly 80% of the length of the bone, giving proximal humeral fractures an almost unrivalled capacity to remodel [13,39]. Consequently, the great majority are treated nonoperatively with a sling or collar-and-cuff, and even severely displaced Neer–Horowitz grade III–IV injuries achieve excellent long-term function without surgery [39,40]. Widely applied thresholds accept angulation of up to about 60° together with near-complete displacement in children younger than approximately 12 years, reducing to about 40° in older children and adolescents whose remodeling reserve is declining [39]. Recent comparative data confirm that operative treatment of displaced fractures offers no clinical or economic benefit over conservative care, and surgery should be reserved for open fractures, neurovascular injury, associated ipsilateral limb fractures, and polytrauma [40]. In the polytraumatized or “floating-limb” child, this threshold, like the region-specific limits tabulated throughout this review, shifts toward operative fixation where stabilization may be required to permit nursing, mobilization, and the care of concomitant injuries. As at the clavicle, the large accepted angles at the proximal humerus presuppose a young child with most growth remaining and reflect in part the wide compensating arc of the shoulder. They should be applied more cautiously as skeletal maturity approaches.

### 5.3. Humeral Shaft

Humeral shaft fractures constitute only 2–5% of childhood fractures and are likewise forgiving, because malunion is well compensated by the wide arc of glenohumeral motion, so that cosmesis rather than function is usually the only residual concern. Most are managed with a hanging cast, coaptation splint, collar-and-cuff, or functional (Sarmiento) brace. Commonly cited acceptable limits are of the order of 20° of varus/valgus and 20° of anterior (procurvatum) angulation, up to 15° of malrotation and 1–2 cm of shortening, with more generous angulation, 20–30°, tolerated in younger children and 15–20° in adolescents. Humeral overgrowth compensates for a moderate degree of bayonet apposition, and the radial-nerve palsy that occasionally accompanies these fractures is almost always a neurapraxia that recovers spontaneously, and it is not in itself an indication for exploration. As with the forearm, the recent drift toward internal fixation is not supported by evidence of better outcomes [41] (Table 2). The rotational and angular limits quoted here are traditional figures derived largely from expert opinion and biomechanical reasoning rather than from comparative trials and are best treated as pragmatic guides (Section Search Strategy and Evidence Appraisal).

## 6. Elbow

### 6.1. Supracondylar Fractures of the Distal Humerus

Supracondylar fractures are the most common elbow fracture of childhood and the region where the conservative window is narrowest, because displaced fractures endanger the brachial artery and median nerve and tend to malunite into cubitus varus. Management follows the modified Gartland classification. Type I fractures, which are non-displaced and have an intact anterior humeral line, are stable and are treated nonoperatively in an above-elbow cast at about 90° of flexion for roughly three weeks [42]. Type II fractures are heterogeneous. Those with an intact anterior cortex and only mild extension can be managed by closed reduction and casting, and long-term follow-up of unreduced type II fractures shows that sagittal-plane malunion remodels well in younger children, whereas unstable type II fractures with rotation or medial comminution, and all type III and IV fractures, require closed reduction and percutaneous pinning [42,43,44]. The reliably conservative supracondylar fracture is therefore type I, with selected stable type II fractures amenable to reduction and cast in the younger child [42,44]. It is important to distinguish the sagittal extension deformity, which remodels reliably in the young child, from coronal malalignment and rotation, which remodel poorly and are the substrate for the cubitus varus that follows an inadequately reduced fracture. The latter therefore justify reduction even when sagittal alignment appears acceptable.

### 6.2. Lateral Humeral Condyle Fractures

The lateral humeral condyle is the second most common elbow fracture and, being intra-articular and subject to the pull of the common extensor origin, is prone to late displacement and non-union. The consensus threshold is clear. Fractures displaced by 2 mm or less with a congruent articular surface are treated conservatively in an above-elbow cast for four to six weeks, with a check radiograph at about one week to detect the secondary displacement that occurs in a minority within the first ten days, whereas fractures displaced by more than 2 mm require reduction and fixation [45,46]. Vigilant follow-up during casting is mandatory, since delayed union and non-union are the principal hazards of under-treatment [45]. Because the true extent of displacement is frequently underestimated on standard anteroposterior and lateral projections, an internal-oblique radiograph should be obtained, as it best profiles the lateral condyle and most reliably demonstrates the articular gap on which the 2 mm threshold depends.

### 6.3. Medial Epicondyle Fractures

The medial epicondyle is an apophyseal traction avulsion, usually produced by a valgus force or an elbow dislocation. Because it is extra-articular and contributes almost nothing to longitudinal growth, its management is dictated by function rather than by remodeling. A large proportion of these injuries, including many with several millimeters of displacement, are treated nonoperatively with a brief period of immobilization followed by early motion, with good functional outcomes even when the fragment heals slightly displaced or by fibrous union. The generally accepted indications for fixation are a fragment incarcerated within the joint, ulnar-nerve dysfunction, and gross valgus instability in the throwing athlete. The precise displacement threshold, commonly quoted between 5 and 15 mm, remains debated [47]. Prolonged immobilization should be avoided, as its duration correlates with slower recovery of elbow motion [48]. In practice, elbow stability, fragment incarceration, ulnar-nerve status, and the functional demands of the throwing athlete weigh more heavily in the decision than any single millimeter threshold.

### 6.4. Radial Neck (Proximal Radius) Fractures

Radial neck fractures account for 5–10% of pediatric elbow fractures and usually follow a valgus load, and because the normal proximal radius carries about 15° of valgus and 5° of anterior tilt, this baseline must be allowed for when measuring angulation. Angulation of up to 30° with translation of less than 50% (or under 3 mm) is widely accepted for nonoperative treatment, with immobilization for one to two weeks followed by early motion, and a systematic review found that range of motion after closed treatment of fractures angulated less than 30° was almost always preserved, with some authors reporting excellent remodeling up to 45° in the young child. Greater angulation or displacement is addressed first by closed reduction, using percutaneous or intramedullary techniques where required, while open reduction is reserved for the irreducible fracture because it carries the highest rates of stiffness, avascular necrosis, and radio-ulnar synostosis [49,50] (Table 3).

## 7. Forearm and Wrist

### 7.1. Diaphyseal (Both-Bone) Forearm Fractures

Diaphyseal “both-bone” forearm fractures account for about 5% of all childhood fractures and are the injury whose operative rate has risen most steeply, yet closed reduction and above-elbow casting remain the gold standard and yield near-universal union with excellent function when alignment is held within accepted limits. Remodeling here is comparatively modest, because the fracture is far from the wrist physis and outside the plane of a hinge joint, so the shaft is less forgiving than the metaphysis, and rotational malalignment in particular corrects poorly and unpredictably [51,52]. As a working guide, angulation of up to about 15° in the middle and distal thirds and up to 10° in the proximal third is accepted in children under 8–10 years, with more generous bayonet apposition tolerated in the very young. A complete both-bone fracture in a child with two or more years of growth remaining remodels even from a bayonet position, whereas the same fracture in an adolescent demands near-anatomical alignment [53,54]. Because loss of position is common, these fractures require close radiographic follow-up, but a systematic review confirms that conservative and operative treatment give equivalent long-term function, so redisplacement within accepted limits should prompt observation rather than reflex surgery [51,52]. Two patterns are unique to the child’s forearm. These are the greenstick fracture, in which one cortex breaks while the other merely bends, and plastic (bowing) deformation, a lasting cortical bend with no visible fracture line. Both are usually reduced and held in a long-arm cast, but diaphyseal bowing remodels less reliably than a metaphyseal fracture, particularly in the older child, so marked bowing should be corrected to restore the radial bow and forearm rotation [55,56]. Refracture is a particular hazard of the forearm. Although the overall pediatric refracture rate is only about 0.5%, it approaches 4% after diaphyseal both-bone fractures and is roughly eight times higher when the original injury was displaced and required reduction, so vigorous activity should be restricted for several weeks after cast removal [57]. One point requires explicit clarification, because the narrative and Table 4 might otherwise appear to conflict. Although older sources suggested that the forearm’s rotational arc can clinically compensate for as much as 45° of malrotation, rotation is difficult to measure reliably on plain radiographs, and a recent systematic review found the evidence underlying all of these angulation and rotation thresholds to be of low quality. Substantial malrotation should therefore be regarded as poorly tolerated and actively minimized rather than accepted as a routine target [51].

### 7.2. Distal Radial Metaphyseal Fractures

Distal radial metaphyseal fractures are among the most common injuries of childhood and, lying immediately proximal to the most active physis of the upper limb, have outstanding remodeling potential [14]. Fully displaced (bayonet) fractures in young children realign completely with growth, and prospective series accept substantial deformity without loss of function, with up to about 20° of angulation with less than 1 cm of shortening in children under 10 years, and around 15° in adolescents [58,59,60,61]. The most important practical message concerns re-manipulation. Rather than repeatedly manipulate a fracture that has re-angulated in cast, the literature supports accepting up to 30° in children under 9 years, 25° between 9 and 12 years, and 20° over 12 years, because these deformities remodel and repeated manipulation adds anesthetic and physeal risk [62]. Quantitative models now allow the expected remodeling of a given malunion to be predicted, supporting the decision to observe [14]. These re-angulation figures apply to sagittal-plane (dorsal or volar) metaphyseal deformity in a child with at least two years of growth remaining and without substantial shortening or complete translation. They are a guide to avoiding repeated manipulation rather than a fixed age-based rule, and the decision should remain individualized to the plane of the deformity, the amount of remaining growth and any accompanying shortening [58,59,60,61]. At the mildest end of the spectrum, the torus (buckle) fracture is intrinsically stable, and the randomized FORCE trial has shown a soft bandage with immediate discharge to be equivalent to rigid casting for pain and function, so these injuries require neither reduction nor routine follow-up radiographs [23].

### 7.3. Distal Radial Physeal Fractures

Fractures of the distal radial physis, usually Salter–Harris type II, are the most frequent physeal injury of the upper limb. They remodel well, and sagittal-plane angulation on the order of 20–30° is acceptable in the younger child, particularly when it lies in the plane of wrist flexion–extension [14,19]. The overriding principle is restraint. A displaced distal radial physeal fracture should be reduced once, gently, and then left, because repeated or late manipulation markedly increases the risk of iatrogenic growth arrest, which although uncommon can produce progressive deformity and warrants follow-up until Park–Harris lines confirm symmetrical growth [19]. Radiographic surveillance every three months until the Park–Harris lines appear parallel is therefore advised [16,19].

### 7.4. Monteggia and Galeazzi Fracture-Dislocations

A Monteggia lesion, a fracture, or plastic deformation of the ulna with dislocation of the radial head accounts for only about 0.4% of pediatric forearm fractures but is disproportionately important, because a missed radial-head dislocation leads to fixed deformity that is difficult to reconstruct [63]. In children the great majority can be treated closed, and management is dictated by the ulnar fracture pattern. Plastic deformation and incomplete (greenstick or buckle) ulnar fractures are treated by closed reduction of the ulnar bow and long-arm casting, which restores ulnar length and alignment and reduces the radial head, whereas complete transverse or oblique ulnar fractures, or a radial head that will not stay reduced, require stabilization of the ulna. The key to conservative success is early diagnosis and restoration of ulnar alignment, with the elbow immobilized in the position that holds the radial head reduced, and residual ulnar angulation under about 10° may itself remodel [63,64]. The much rarer Galeazzi injury, a distal radial fracture with disruption of the distal radio-ulnar joint, is likewise usually managed in the child by closed reduction and immobilization [65] (Table 4). Because a missed radial-head dislocation is the single decisive failure mode, early recognition being the difference between straightforward conservative success and difficult, often unsatisfactory, late reconstruction, the radiocapitellar line, which in every projection should pass through the center of the capitellum, must be verified on the initial radiographs of every forearm fracture and again after reduction, and any ulnar bow or plastic deformation should prompt a deliberate search for radial-head malalignment [63,64].

**Table 4:** Acceptable alignment for conservative treatment of pediatric forearm, distal radius, and Monteggia injuries.

Fracture (Location)	Age/Sex	Acceptable Deformity for Conservative Treatment	Ref.
Diaphyseal both-bone, mid/distal third	Girls <8/boys <10 y	~15° angulation. Bayonet apposition. Malrotation corrects poorly and should be minimized	[53,65]
Diaphyseal both-bone, proximal third	Girls <8/boys <10 y	≤10° angulation (stricter than distal)	[51,65]
Diaphyseal both-bone	≥2 y growth remaining	Bayonet apposition with ~10–15° angulation; minimal malrotation	[53,54]
Diaphyseal both-bone	>9 y	~10° (proximal) to 15° (distal) angulation; malrotation corrects poorly and should be minimized	[65]
Distal radial metaphysis	<10 y	Up to ~20° angulation; <1 cm shortening; complete displacement remodels	[59,60]
Distal radial metaphysis (re-angulated in cast)	<9/9–12/>12 y	Accept 30°/25°/20° rather than re-manipulate	[62]
Distal radius	Adolescent (girls 11–14/boys 13–15 y)	~15° angulation	[58]
Distal radial physis (Salter–Harris I/II)	<10–12 y	~20–30° sagittal angulation; reduce once, avoid repeat manipulation	[14,19]
Monteggia—plastic or greenstick ulna	4–10 y	Closed reduction of ulnar bow + long-arm cast; radial head reduces with the ulna	[63,64]

The evidence behind these limits is uneven. The nonoperative management of the distal radial buckle (torus) fracture rests on high-quality randomized evidence (the FORCE trial), and recent systematic-review and meta-analysis data inform the diaphyseal angulation criteria, whereas the age-stratified angulation values derive largely from older series and traditional practice, and the rotational (malrotation) limits rest on low-quality evidence and are difficult to measure on plain radiographs. Each value should be read as a clinical guide rather than a fixed cut-off (see Section Search Strategy and Evidence Appraisal).

## 8. Hand

Hand fractures are among the most common injuries of later childhood, and the great majority are managed nonoperatively, because a thick periosteum and adjacent physes confer good remodeling and rigid anatomical reduction is seldom necessary for function [66]. The cardinal rule at the hand is that rotational malalignment and scissoring of the fingers must never be accepted, since these are judged clinically in flexion, not on the radiograph, whereas pure sagittal angulation is generally forgiving [66,67].

### 8.1. Metacarpal Fractures

Metacarpal fractures most often involve the neck of the little finger (the ‘boxer’s fracture’), and the tolerance for apex-dorsal angulation increases from the radial to the ulnar side because of the greater compensatory mobility of the ulnar carpometacarpal joints, with roughly 10° accepted in the index, 20° in the long, 30° in the ring, and 40° in the little metacarpal, with even more tolerated in the young child whose distal metacarpal physis is still open [66,68]. Most are treated by closed reduction and a short period of immobilization, with surgery being reserved for clinical malrotation, open injuries, and irreducible displacement [68,69]. Shaft fractures tolerate less angulation than neck fractures and demand particular attention to rotation [68].

### 8.2. Phalangeal Fractures

Phalangeal fractures follow the same principles. Sagittal-plane angulation of the proximal and middle phalanges remodels with growth and is well tolerated, but any coronal angulation or malrotation must be corrected [66,67]. Two patterns are exceptions that should not be treated as ordinary conservative fractures. The phalangeal neck and displaced condylar fractures are unstable and usually require reduction and pinning, and the Seymour fracture (an open physeal fracture of the distal phalanx with the nail plate interposed in the fracture) requires irrigation, extraction of the interposed tissue, reduction, and fixation to avoid infection and growth arrest. A late-presenting condylar fracture that is well aligned in the coronal plane may still be observed, since sagittal malalignment can remodel even in the older child [66].

### 8.3. Carpal (Scaphoid) Fractures

Carpal fractures are dominated by the scaphoid, which is rare before the age of ten and occurs chiefly in adolescents. Historically, the fracture lies at the distal pole and is minimally displaced, and more than 90% unite with cast immobilization, the non-union rate being under about 1.5% and largely confined to missed or late-presenting injuries [70,71]. A non-displaced scaphoid fracture is treated in a below-elbow cast, thumb-spica by tradition, though wrist immobilization alone appears equally effective, until union, which is quickest at the distal pole and slowest at the proximal pole. Displaced fractures, proximal-pole fractures at risk of avascular necrosis and established non-unions require internal fixation, sometimes with bone grafting [71] (Table 5).

## 9. Pelvis and Hip

### 9.1. Pelvic Ring and Apophyseal Avulsion Fractures

The pediatric pelvis behaves very differently from the adult. Its greater cartilaginous content and elasticity allow it to absorb energy, so that most pelvic ring injuries in children are stable and are managed nonoperatively with a short period of protected weight-bearing followed by rehabilitation. Operative stabilization is reserved for the uncommon unstable ring disruption, usually in the setting of major trauma and hemodynamic instability [72,73]. The commonest pediatric pelvic injury is the apophyseal avulsion, occurring at the anterior superior and inferior iliac spines, the ischial tuberosity, the iliac crest, and the trochanters, produced by a violent muscle contraction in an adolescent athlete [72]. These are almost always treated conservatively with rest, analgesia, protected weight-bearing for four to six weeks, and graded rehabilitation, and a systematic review shows that conservative and operative treatment give comparable long-term outcomes. Operative fixation is considered only for large displacement, generally more than 1.5–2 cm, and particularly at the ischial tuberosity where wider displacement predisposes to painful non-union, or for the competitive athlete seeking the fastest return to sport [72,73].

### 9.2. Femoral Neck (Proximal Femoral) Fractures

The femoral neck is the counter-example to almost every other region in this review, because the blood supply to the immature femoral head is precarious, a displaced femoral neck fracture is an orthopedic emergency, and avascular necrosis is the dominant complication. Fractures are described by the Delbet classification, and the risk of avascular necrosis rises the more proximal the fracture, being highest in the rare transepiphyseal (type I) injury and progressively lower in transcervical (II), cervicotrochanteric (III), and intertrochanteric (IV) fractures. For this reason the great majority of displaced femoral neck fractures require urgent, anatomical reduction and internal fixation, often with capsular decompression, rather than conservative care [74,75]. The role of purely conservative treatment is correspondingly narrow. A genuinely undisplaced fracture, or an intertrochanteric fracture in a young child, may be held in a hip spica, but even undisplaced fractures carry a substantial risk of secondary displacement in cast, approaching half in some series, so spica treatment demands very close radiographic surveillance and a low threshold for conversion to fixation [74]. The hip is thus the region where the balance tips decisively toward surgery, and conservative management is the exception reserved for the youngest children with stable, undisplaced patterns [74,75] (Table 6).

## 10. Femur

### 10.1. Femoral Shaft (Diaphyseal) Fractures

The femoral shaft epitomizes age-dependent, remodeling-driven management. Isolated shaft fractures unite rapidly, tolerate considerable deformity and reliably overgrow by up to about 1 cm, so that moderate shortening is not merely accepted but expected to compensate over the following years. Treatment is therefore chosen chiefly by age and weight rather than by fracture pattern. In infants younger than about six months, a Pavlik harness is the treatment of choice. Between roughly six months and five years, early closed reduction and a hip spica cast is the gold standard for isolated low-energy fractures with less than 2 cm of initial shortening. From about five to eleven years, children are increasingly managed with flexible (elastic) intramedullary nails. The overlap around five to six years is a genuine transition zone in which body size and weight, rather than age alone, guide the choice. Heavier adolescents are treated with rigid nails or submuscular plates, yet even in these groups conservative treatment remains legitimate when the fracture is stable. Acceptable alignment tightens with age. In the youngest children as much as 20–30° of angulation and 2–3 cm of shortening will remodel, narrowing to roughly 15° of varus/valgus, 20° in the sagittal plane, and 15 mm of shortening in mid-childhood, and to about 5–10° with 1 cm of shortening in the adolescent, whereas rotational malalignment remodels negligibly and is not accepted at any age. These age-stratified figures are practical treatment tolerances applied at reduction and casting rather than measured post hoc remodeling limits. The shortening allowances in particular are set to exploit the predictable post-fracture overgrowth of the femur, while the angulation allowances anticipate remodeling in the plane of hip and knee motion, so both should be read as guides to what may safely be left uncorrected in the cast rather than as endpoints to be aimed for [76,77,78,79,80,81,82]. The principal pitfalls of spica treatment are excessive shortening, predicted by a positive intra-operative ‘telescope’ test and by more than 2 cm of initial overlap, and skin complications, so spica-treated children must be followed radiographically during the first weeks [76,77]. Femoral overgrowth after a shaft fracture averages roughly 0.6–1 cm and exceeds 1 cm in about a quarter of children, driven by post-fracture hyperaemia and largely ceasing by 18 months, which is why a shortened, slightly overriding position is deliberately accepted in the young child. Where flexible intramedullary nails are used in the school-aged child, a length-unstable fracture pattern predisposes to shortening and nail-end irritation [78,79]. Flexible intramedullary nailing, introduced by the Nancy group in 1988, has become the commonest fixation between about five and eleven years, though a body weight above 50 kg predicts nail-related complications, and where a shaft fracture nonetheless unites with angulation, remodeling in the plane of hip and knee motion reliably corrects it in the younger child [80,81,82].

### 10.2. Distal Femoral Physeal Fractures

The distal femoral physis is the exception at the knee. Although Salter–Harris I and II fractures elsewhere carry a low risk of growth disturbance, the distal femoral physis is uniquely vulnerable, since physeal bar formation and growth arrest follow in roughly a third to a half of cases, and the risk rises with the degree of displacement. For this reason even minimally displaced fractures are followed closely, and any displaced fracture is reduced anatomically and stabilized, usually operatively, rather than left in a cast, because a long-leg cast alone frequently fails to hold the reduction. Purely conservative treatment is therefore confined to genuinely non-displaced fractures, and every distal femoral physeal injury, however treated, requires surveillance until Park–Harris lines confirm symmetrical growth, since a partial arrest produces progressive angular deformity or limb-length discrepancy that may itself demand later surgery [83] (Table 7).

## 11. Knee and Leg

### 11.1. Patella, Tibial Eminence, and Tibial Tubercle

The knee and leg span the full spectrum from injuries that must be reduced anatomically to fractures that are almost always conservative. Three peri-articular injuries turn on the integrity of the joint surface and the extensor mechanism. Patellar fractures are rare in children and are usually “sleeve” avulsions, and a non-displaced fracture with an intact extensor mechanism is treated in a cylinder cast or knee immobilizer in extension, whereas a displaced fracture with a disrupted extensor mechanism requires fixation [84]. Tibial eminence (anterior cruciate avulsion) fractures are governed by their displacement. Non-displaced (Meyers–McKeever type I) and reducible hinged (type II) fractures are treated by aspiration and immobilization in near-extension, while completely displaced (type III/IV) fractures are fixed, because conservative treatment of a displaced eminence leads to non-union, residual laxity, and loss of extension [85]. The adolescent tibial tubercle fracture is likewise displacement-dependent. Minimally displaced extra-articular fractures are held in a cylinder cast in extension, but most displaced or intra-articular patterns require screw fixation of the extensor mechanism [86]. In each of these three peri-articular injuries the conservative window is defined not by an angular threshold but by two anatomical criteria, restoration of articular congruity and integrity of the extensor mechanism, so that a minimally displaced fracture with a congruent joint surface and a competent extensor apparatus may be treated in a cast, whereas any displacement leaving an articular step-off or an incompetent extensor mechanism mandates fixation [84,85,86].

### 11.2. Proximal Tibial Metaphyseal Fractures

The proximal tibial metaphyseal fracture demonstrates both remodeling and its limits. These fractures are treated in a long-leg cast, but they are notorious for developing a progressive valgus deformity (the Cozen phenomenon) in the months after union, even when the initial reduction was perfect. The essential message is that this valgus usually corrects spontaneously by remodeling over one to three years and should be observed rather than corrected surgically, since early osteotomy tends to recur, and the family should be warned of the deformity at the outset [4,87]. The related proximal tibial “trampoline” fracture of the toddler is managed conservatively and likewise remodels, although the correction may be incomplete [87]. Although spontaneous correction is the rule, it is not invariable. A mechanical-axis deviation that persists beyond about two to three years, or a valgus that remains symptomatic, may warrant temporary hemi-epiphyseal tethering (guided growth), which reliably restores the axis and has largely replaced corrective osteotomy for this indication [88].

### 11.3. Tibial and Fibular Shaft Fractures

Tibial shaft fractures account for around 15% of pediatric fractures and are, in most children, a conservative injury. Closed reduction and a well-molded cast that controls length, alignment, and rotation gives excellent results. Accepted limits are of the order of less than 10° of angulation in both the coronal and sagittal planes, less than 50% translation and under 1 cm of shortening, although many surgeons apply the stricter criteria of under 5° of varus/valgus and under 10° of recurvatum. An isolated tibial fracture with an intact fibula tends to drift into varus and must be watched closely and wedged if necessary during the first three weeks, whereas a below-knee cast with early weight-bearing is sufficient for many isolated fractures. Varus and valgus should not be regarded as equivalent. With an intact fibula the tibia drifts predictably into varus, which remodels less reliably than physiological valgus and is therefore the deformity to guard against, while rotational malalignment at the tibia remodels poorly and is better corrected at the time of casting than accepted. The “toddler’s fracture”, an undisplaced spiral fracture of the tibia in the ambulant infant, needs only a below-knee cast, or according to recent work sometimes merely a supportive bandage, and heals within three to four weeks [89,90]. Surgery is reserved for open, comminuted, or unstable fractures, those with compartment syndrome or vascular injury, and the older adolescent in whom remodeling can no longer be relied upon [89] (Table 8).

## 12. Ankle and Foot

### 12.1. Ankle: Distal Tibial and Fibular Physeal Fractures

Ankle fractures are the second most common physeal injuries of childhood. The distal fibula is usually injured through a Salter–Harris I or II pattern or as a lateral-malleolar avulsion, and these stable injuries are treated in a below-knee walking cast or a removable boot with reliable union. Distal tibial Salter–Harris I and II fractures are reduced closed and immobilized, tolerating a few degrees of residual angulation in the younger child, but they must be followed for growth disturbance, which is more common here than at most physes [19]. The transitional fractures of the adolescent, namely the juvenile Tillaux (a Salter–Harris III of the anterolateral tibial epiphysis) and the triplane fracture, are intra-articular, and a computed tomogram is used to measure the true articular gap because plain films underestimate it [91]. The widely applied rule treats fractures with 2 mm or less of articular displacement in a cast and reduces and fixes those displaced by more than 2 mm to restore joint congruity. Comparative series show that conservatively and operatively treated fractures within this framework achieve similarly good outcomes, and recent data even question whether gaps of 2–5 mm truly require surgery [91,92]. This emerging evidence is of interest but does not yet justify abandoning the established principle of articular congruity. The studies are retrospective and small, a residual gap remained a negative prognostic factor even after operative treatment, and anatomical reduction therefore remains the safer default for a displaced intra-articular fragment [92]. The computed tomography that guides these decisions carries a radiation cost that is not trivial in a child, so it should be reserved for fractures in which the result will change management, and a low-dose or limited-slice protocol should be used where available. Isolated undisplaced distal fibular Salter–Harris I and II and avulsion fractures are the most common lower-limb fractures of childhood and among the lowest-risk. Many radiograph-negative injuries once labeled Salter–Harris I prove on MRI to be ligamentous sprains, and these low-risk injuries recover as well in a removable brace or walking boot as in a cast, permitting earlier return to activity [93,94,95]. Magnetic-resonance studies confirm that most such radiograph-negative injuries in fact spare the growth plate and are ligamentous, supporting brief symptomatic immobilization over prolonged casting [95,96].

### 12.2. The Foot: Metatarsals, Phalanges, Calcaneus, and Talus

The child’s foot is remarkably forgiving. Foot fractures make up 5–13% of pediatric fractures, half of them in the metatarsals and phalanges, and the great majority heal well with nonoperative care [97]. Metatarsal shaft fractures are treated in a short-leg walking cast, and even substantial translation remodels and unites without symptoms, and operative treatment is reserved for the older adolescent with multiple fractures or marked displacement, and for the first metatarsal [98]. The base of the fifth metatarsal is treated in a cast, with the caveat that the proximal-diaphyseal (Jones) fracture has a tenuous blood supply and is prone to non-union and refracture, so fixation is considered in the older athletic adolescent. Toe phalangeal fractures need only buddy-strapping or a rigid-soled shoe. The hindfoot is different. Talar and calcaneal fractures are rare in children, and while non-displaced fractures do well in a cast, particularly in the child under eight, whose cartilaginous anlage heals readily, a displaced intra-articular fracture of the talus, with its risk of avascular necrosis, or of the calcaneus in the older child warrants anatomical reduction and fixation [97] (Table 9).

## 13. When Conservative Treatment Fails: Indications for Surgery

Nothing in this review argues against surgery where surgery is genuinely indicated, and the aim has been to define the boundary, not to erase it. Across every region a small set of circumstances mandates operative treatment irrespective of the child’s remodeling potential. The absolute indications are consistent. They are the open fracture, which requires debridement and stabilization, and the fracture complicated by vascular injury or an evolving compartment syndrome. They also include the displaced intra-articular fracture, in which remodeling cannot restore joint congruity, such as the displaced lateral humeral condyle, the Tillaux and triplane fractures, and the tibial eminence and tubercle. Finally, they include the irreducible fracture, usually owing to soft-tissue interposition, and the fracture that simply cannot be held within acceptable limits in a cast [5,12]. The open fracture deserves a particular comment. Prompt intravenous antibiotics and tetanus prophylaxis are the immediate priority, and while Gustilo–Anderson type II and III injuries require formal operative irrigation, debridement, and stabilization, a growing body of pediatric evidence shows that many type I open fractures, most of which involve the forearm, can be managed with early antibiotics, bedside irrigation, and closed reduction without a visit to theatre, with infection rates at or below about 3%. This nonoperative pathway is appropriate only for a carefully selected, low-energy type I wound without gross contamination, devitalized tissue, neurovascular injury, compartment syndrome, or an unstable fracture configuration. Early antibiotics remain mandatory, and the decision should follow institutional guidelines and specialist assessment rather than being made ad hoc [99,100,101,102].

Certain physeal injuries also demand anatomical reduction and fixation because malreduction produces a growth-arresting bar, most importantly the displaced distal femoral physeal fracture and displaced Salter–Harris III and IV injuries, while the displaced femoral neck fracture is treated as an emergency because of the precarious blood supply to the femoral head [74,83,91]. Beyond these, a set of relative indications describes situations in which operative treatment is reasonable but not obligatory. These include the polytraumatised child or the ‘floating’ limb with fractures above and below a joint, in whom fixation simplifies nursing and early mobilization, the heavier adolescent approaching skeletal maturity, in whom remodeling can no longer be relied upon, and the unstable diaphyseal forearm or tibial fracture in the older child, where fixation reduces the risk of redisplacement [5,12,52]. The essential discipline is to separate these genuine indications from the reflex to operate on a deformity that the child’s own physis would have corrected [3,5,13] (Table 10). Two clarifications refine this grading. Failure to hold alignment is the one category that resists tidy classification. It becomes an absolute indication once acceptable alignment genuinely cannot be maintained despite an adequate cast and one appropriate re-manipulation but remains relative while further reasonable attempts at closed control are still available. And because the polytraumatised or ‘floating’-limb child is a recurring modifier rather than a single-region problem, the region-specific thresholds tabulated throughout this review should all be read as shifting toward fixation when injuries are multiple or when operative stabilization is needed to permit nursing, mobilization, and the care of other injuries.

## 14. Synthesis and Clinical Algorithm

The clinical consequences of a physeal injury vary greatly with its location. Table 11 draws together the growth plates at greatest risk, namely the distal femoral, distal tibial, distal radial, and proximal humeral physes, with the Salter–Harris patterns of concern, the approximate risk of growth arrest, the circumstances that call for anatomical reduction, a sensible surveillance interval, and the interpretation of the Park–Harris line (Table 11). It follows that the displaced femoral neck fracture and the highest-risk physeal injuries, above all those of the distal femur, are the principal exceptions to the conservative philosophy of this review. For these, operative treatment is the default and nonoperative management is appropriate only in exceptional circumstances, so that the strong presumption in favor of conservative care that runs through the rest of this review should be deliberately reversed [3,13].

**Figure 1:**
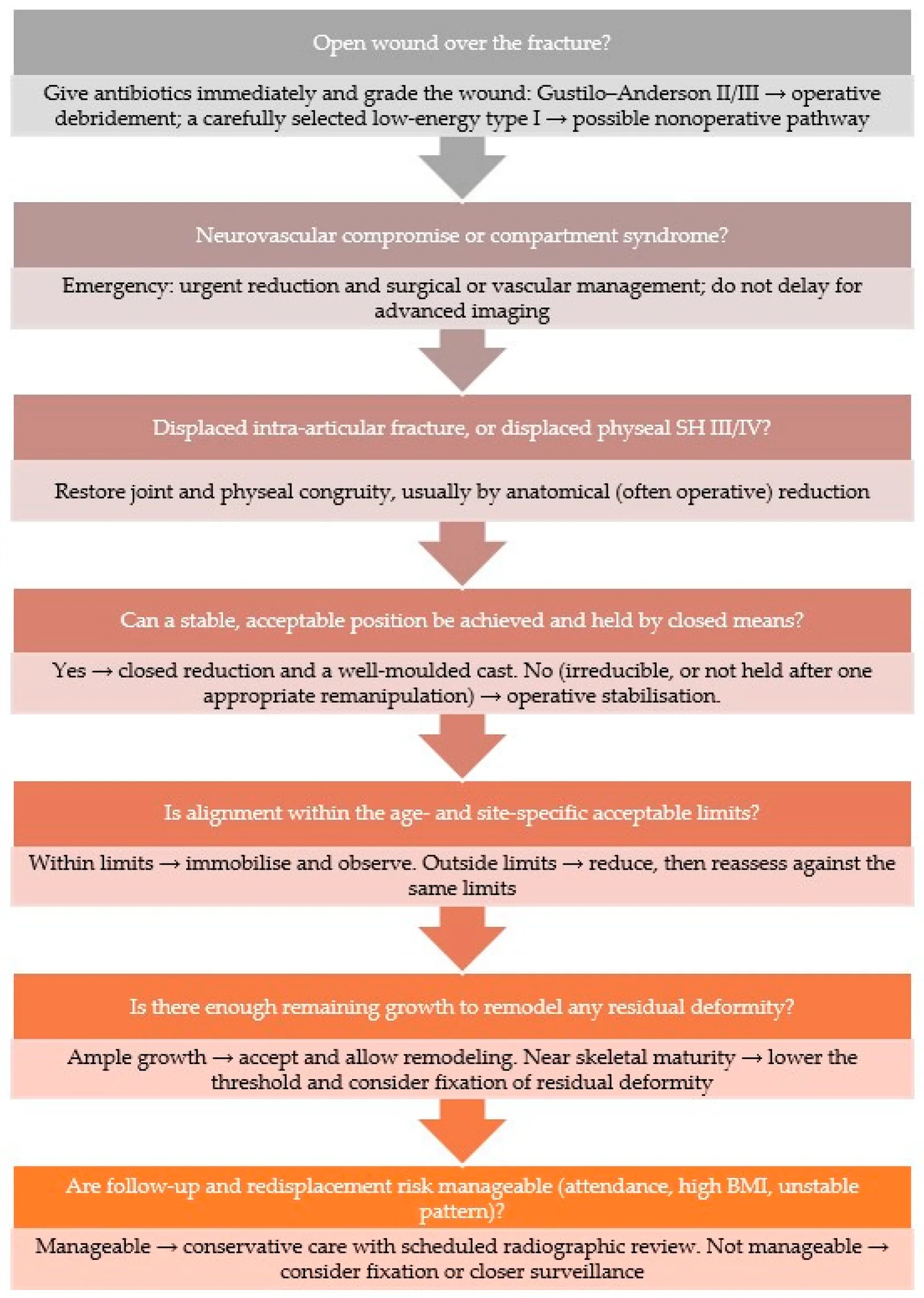
A stepwise algorithm for the initial management of a pediatric fracture. Steps are applied in order; a positive finding at any of the first three steps generally overrides the remodeling considerations that follow.

Translating these principles into practice benefits from an explicit sequence. Figure 1 sets out a stepwise algorithm that moves from the injuries mandating urgent or operative care, open wounds, neurovascular compromise, and displaced intra-articular or high-risk physeal fractures, through reducibility and the age- and site-specific alignment limits, to the questions of remaining growth and the practicality of follow-up. It is intended to structure, not replace, clinical judgment.

## 15. Conclusions

The immature skeleton is the most forgiving tissue in trauma surgery. From the mandibular condyle to the phalanges of the foot, a thick periosteum, an active physis, and a powerful remodeling response allow the great majority of children’s fractures to be treated without an operation, provided the deformity lies within limits that growth will correct. Those limits are neither arbitrary nor uniform. They are region-specific, age-specific, and plane-specific, and this review has gathered them, region by region. The recurring lessons are constant. Angulation in the plane of the neighboring joint’s motion remodels, coronal-plane angulation remodels less, and rotation and intra-articular incongruity remodel poorly and unpredictably. The younger the child and the closer the fracture to an active physis, the more deformity may safely be accepted, while the capacity to remodel tracks the activity of the nearest physis and the plane of the deformity rather than any simple proximal-to-distal or lower-versus-upper-limb rule. Repeated manipulation is rarely justified for a deformity that will correct spontaneously, and it carries its own anesthetic and physeal costs.

Against this biological backdrop, the documented drift toward operative fixation of injuries that would have remodeled should be critically evaluated, particularly where remodeling potential is well documented and no durable advantage of surgery has been demonstrated, since it may expose children to the avoidable hazards of anesthesia, infection, implant complications, and reoperation. Surgery retains a clear and essential role, namely for open, intra-articular, irreducible, unstable and high-risk physeal fractures, for the displaced femoral neck, and for the adolescent near maturity, but it should be chosen because a genuine indication is present, not because a radiograph is imperfect or because remodeling has been forgotten. For the clinician who treats children, a secure command of the acceptable thresholds set out here is the surest protection against both under- and over-treatment, and the best guarantee that the extraordinary healing capacity of the child’s skeleton is used to the child’s advantage. The region-specific thresholds gathered here are clinical guides that inform, but cannot replace, an individualized assessment of each child.

## Figures and Tables

**Table 1:** Conservative management of craniofacial and spinal fractures in children.

Region/Fracture	Preferred Conservative Approach	Acceptability and Remodeling	Ref.
Mandibular condyle	Soft diet ± brief maxillomandibular fixation; functional orthodontic appliances	Remodels even with >25° angulation or >9 mm displacement if the head remains in the fossa; open reduction risks growth arrest	[31,32,33]
Thoracolumbar compression	Analgesia, early mobilization, TLSO brace 6–8 weeks	Stable, posterior ligamentous complex intact, no neurological deficit; residual wedge partly remodels	[34,35]
Stable burst	TLSO/body brace	Acceptable if low canal retropulsion and neurologically intact	[34,35]
Unstable spine (Chance, PLC disruption, marked retropulsion, deficit)	Not suitable—surgical stabilization	Conservative treatment contraindicated	[34,35]

The evidence behind these recommendations is uneven, and this section is intended as a focused overview. The favorable remodeling of the pediatric mandibular condyle and the conservative management of stable thoracolumbar compression fractures are well recognized, but the condylar remodeling figures derive from selected extracapsular fractures rather than from every pattern, and much of the spinal guidance rests on smaller series and on expert consensus extrapolated in part from adult practice. Each value should be read as a clinical guide rather than a fixed cut-off (see Section Search Strategy and Evidence Appraisal).

**Table 2:** Acceptable alignment for conservative treatment of clavicle and upper-arm fractures in children.

Fracture	Age	Acceptable Deformity (Angulation/Rotation/Shortening/Displacement)	Ref.
Clavicle, mid-shaft	All pediatric ages	Complete displacement, shortening, and angulation acceptable; remodeling of up to ~90° angulation and 4 cm overlap reported	[36,37,38]
Proximal humerus	<12 y	Up to ~60° angulation with near-complete displacement	[39]
Proximal humerus	≥12 y/adolescent	Up to ~40° angulation	[39,40]
Humeral shaft	Younger child (<12 y)	20–30° angulation; ≤15° rotation; 1–2 cm shortening; bayonet apposition	[41]
Humeral shaft	Adolescent	15–20° angulation; ≤15° rotation; 1–2 cm shortening	[41]

The evidence behind these limits is uneven. Nonoperative management of clavicular and proximal humeral fractures is supported by consistent contemporary outcome data, but the large accepted angles reflect functional compensation at the mobile shoulder girdle as much as true osseous remodeling, and the humeral-shaft angular and rotational figures derive largely from traditional practice and expert opinion. Each value should be read as a clinical guide rather than a fixed cut-off (see Section Search Strategy and Evidence Appraisal).

**Table 3:** Acceptable criteria for conservative treatment of elbow fractures in children.

Fracture	Conservative Window	Threshold for Surgery	Ref.
Supracondylar (distal humerus)	Gartland I and selected stable type II—above-elbow cast ~90° flexion, ~3 wk	Unstable type II, III, IV; vascular or nerve compromise	[42,43,44]
Lateral humeral condyle	≤2 mm displacement, congruent joint—cast 4–6 wk + 1-wk check film	>2 mm displacement; late displacement/non-union	[45,46]
Medial epicondyle	Most, including several-mm displacement—brief immobilization, early motion	Fragment incarcerated in joint; ulnar-nerve dysfunction; valgus instability (throwers)	[47,48]
Radial neck (proximal radius)	≤30° angulation and <50% (or <3 mm) translation—immobilize 1–2 wk	>30–45° angulation or >50% translation; irreducible fracture	[49,50]

The evidence behind these criteria is uneven. The spontaneous correction of sagittal-plane supracondylar deformity is well recognized, whereas the operative thresholds for the lateral condyle, medial epicondyle, and radial neck rest largely on retrospective series and expert opinion (for the medial epicondyle in particular, elbow stability, fragment incarceration, and ulnar-nerve status are more informative than any single displacement figure). Each value should be read as a clinical guide rather than a fixed cut-off (see Section Search Strategy and Evidence Appraisal).

**Table 5:** Acceptable criteria for conservative treatment of hand and carpal fractures in children.

Fracture	Detail	Acceptable Deformity for Conservative Treatment	Ref.
Metacarpal neck (boxer’s)	Index/long/ring/little	~10°/20°/30°/40° apex-dorsal angulation; more if physis open; no malrotation	[66,68]
Metacarpal shaft	—	Less tolerant than neck; correct malrotation; closed if angulation modest and no rotation	[68,69]
Proximal/middle phalanx	—	Sagittal angulation remodels; no coronal angulation or malrotation accepted	[66,67]
Phalangeal neck/displaced condylar	—	Unstable—usually reduction and pinning; well-aligned late condylar may be observed	[66]
Seymour (open distal phalangeal physis)	—	Not conservative—irrigation, extraction, reduction, fixation	[66]
Scaphoid, non-/minimally displaced	Distal pole or waist, adolescent	Short-arm (± thumb) cast to union; >90% unite	[70,71]
Scaphoid displaced/proximal pole/non-union	—	Not conservative—fixation ± bone graft	[71]

The evidence behind these limits is uneven. The tolerance of metacarpal-neck angulation and the operative management of Seymour and of displaced or proximal-pole scaphoid fractures are well recognized, whereas the metacarpal and phalangeal angular and rotational limits derive largely from expert opinion. Each value should be read as a clinical guide rather than a fixed cut-off (see Section Search Strategy and Evidence Appraisal).

**Table 6:** Conservative treatment of pelvic and hip fractures in children.

Fracture	Conservative Approach	When to Operate	Ref.
Pelvic apophyseal avulsion (ASIS, AIIS, ischial tuberosity, crest, trochanters)	Rest, protected weight-bearing 4–6 wk, graded rehabilitation	Displacement > 1.5–2 cm, or competitive athlete	[72,73]
Stable pelvic ring (no instability)	Protected weight-bearing, mobilize as pain allows	Unstable ring disruption/hemodynamic instability	[72,73]
Femoral neck, undisplaced (young child; Delbet III/IV)	Hip spica with very close radiographic follow-up	~50% risk of displacement in cast—low threshold to fix	[74]
Femoral neck, displaced (Delbet I–IV)	Not conservative—urgent reduction + internal fixation ± capsulotomy	AVN is the dominant risk, highest in type I	[74,75]

The evidence behind these recommendations is uneven. Nonoperative treatment of stable pelvic-ring and apophyseal avulsion injuries and the operative management of the displaced femoral neck fracture are well established, whereas the displacement figure for apophyseal avulsion derives largely from retrospective series and expert opinion. Each value should be read as a clinical guide rather than a fixed cut-off (see Section Search Strategy and Evidence Appraisal).

**Table 7:** Acceptable alignment and treatment of pediatric femoral fractures.

Fracture/Age	Preferred Management	Acceptable Deformity/Note	Ref.
Femoral shaft, <6 months	Pavlik harness	Marked overgrowth compensates; rotation not accepted	[76,77]
Femoral shaft, 6 mo—5/6 y	Early hip spica (isolated, <2 cm shortening)	Up to ~20–30° angulation and 2–3 cm shortening remodel in the youngest	[76,77]
Femoral shaft, ~2–10 y	Spica or flexible IM nail	~10–15° varus/valgus; 15–20° sagittal; ≤15 mm shortening	[76,77]
Femoral shaft, adolescent	Flexible/rigid nail or plate	~5–10° angulation; ≤10 mm shortening; no rotation	[76,77]
Distal femoral physis, non-displaced	Cast with very close follow-up	Growth arrest in ~30–50%; risk rises with displacement	[83]
Distal femoral physis, displaced	Not conservative—anatomical reduction + fixation	Long-leg cast alone fails to hold; high arrest risk	[83]

The evidence behind these values is uneven. The age-stratified femoral-shaft angulation and shortening figures are pragmatic treatment tolerances derived largely from traditional practice and older series rather than measured remodeling limits, whereas the high risk of growth arrest after displaced distal-femoral physeal injury is well documented. Each value should be read as a clinical guide rather than a fixed cut-off (see Section Search Strategy and Evidence Appraisal).

**Table 8:** Acceptable criteria for conservative treatment of knee and leg fractures in children.

Fracture	Conservative Window	Surgery/Caveat	Ref.
Patella (sleeve avulsion)	Non-displaced, extensor mechanism intact—cylinder cast/immobilizer in extension	Displaced with disrupted extensor mechanism—fixation	[84]
Tibial eminence (ACL avulsion)	Type I/reducible type II—aspiration + cast near extension	Type III/IV displaced—fixation	[85]
Tibial tubercle	Minimally displaced extra-articular—cylinder cast in extension	Displaced or intra-articular—screw fixation	[86]
Proximal tibial metaphysis	Long-leg cast; observe	Post-traumatic valgus (Cozen) usually remodels over 1–3 y—avoid early osteotomy	[4,87]
Tibial shaft	Closed reduction + well-molded cast	<10° angulation (coronal & sagittal); <50% translation; <1 cm shortening	[89]
Toddler’s fracture	Below-knee cast (or supportive bandage)	Heals in 3–4 weeks	[89,90]

The evidence behind these criteria is uneven. The spontaneous correction of post-traumatic (Cozen) proximal-tibial valgus and the operative management of displaced extensor-mechanism and intra-articular injuries are well recognized, whereas the tibial-shaft angulation, translation, and shortening limits derive largely from traditional practice and expert opinion. Each value should be read as a clinical guide rather than a fixed cut-off (see Section Search Strategy and Evidence Appraisal).

**Table 9:** Acceptable criteria for conservative treatment of ankle and foot fractures in children.

Fracture	Conservative Window	Surgery/Caveat	Ref.
Distal fibula (SH I/II, avulsion)	Below-knee cast or walking boot	Reliable union; rarely operative	[19]
Distal tibia (SH I/II)	Closed reduction + cast; a few degrees residual accepted in the young	Follow for growth arrest; SH III/IV or displaced—fix	[19,91]
Tillaux/triplane (transitional)	≤2 mm articular gap—cast (CT to measure)	>2 mm—reduction + screw fixation	[91,92]
Metatarsal shaft	Short-leg walking cast	Even marked translation unites; fix if older/multiple/1st MT	[98]
Fifth metatarsal base	Cast	Jones (proximal diaphysis) prone to non-union—consider fixation in athletes	[97]
Toe phalanges	Buddy-strapping/rigid-soled shoe	—	[97]
Talus/calcaneus	Non-displaced—cast (heals well, esp. <8 y)	Displaced intra-articular—fixation (AVN risk)	[97]

The evidence behind these criteria is uneven. The reliable union of distal-fibular and most metatarsal fractures and the operative management of displaced transitional (Tillaux/triplane) and displaced intra-articular talar and calcaneal injuries are well recognized, whereas the 2 mm articular threshold for transitional fractures and the foot-fracture tolerances derive largely from traditional practice and expert opinion. Each value should be read as a clinical guide rather than a fixed cut-off (see Section Search Strategy and Evidence Appraisal).

**Table 10:** Genuine indications for operative treatment of pediatric fractures.

Indication	Type	Ref.
Open fracture (Gustilo–Anderson II/III)	Absolute (selected type I may be nonoperative)	[5,12]
Vascular injury or compartment syndrome	Absolute	[5,12]
Displaced intra-articular fracture (lateral condyle; Tillaux/triplane; tibial eminence/tubercle)	Absolute	[85,91]
Irreducible fracture (soft-tissue interposition)	Absolute	[5]
Displaced high-risk physeal fracture (distal femur; Salter–Harris III/IV)	Absolute	[83,91]
Displaced femoral neck fracture	Absolute (emergency)	[74]
Failure to hold acceptable alignment in a cast	Relative. Absolute once alignment cannot be held despite adequate casting	[12,52]
Polytrauma, floating joint or multiple fractures	Relative	[5]
Adolescent near skeletal maturity (limited remodeling)	Relative	[5,12]
Unstable diaphyseal forearm or tibia in the older child	Relative	[52,89]

The strength of the evidence behind these indications is uneven. The absolute indications, open fracture, vascular injury or compartment syndrome, irreducible fracture, displaced intra-articular and displaced high-risk physeal fractures, and the displaced femoral neck fracture, are well established, whereas the relative indications rest more on individualized judgment and expert consensus than on comparative evidence. Each should be read as a clinical guide rather than a fixed rule (see Section Search Strategy and Evidence Appraisal).

**Table 11:** High-risk physes. Patterns of concern, approximate risk of growth arrest, indications for anatomical reduction, and recommended surveillance.

Growth Plate	SH Types of Main Concern	Approx. Risk of Growth Arrest	When Anatomical Reduction is Indicated	Recommended Surveillance	Significance of the Park–Harris Line
Distal femur	I–IV (the highest-risk physis in the body)	High (~30–50%)	Any displaced SH I/II that cannot be held, and all displaced SH III/IV	Every 3–6 months for 18–24 months	A transverse line resuming parallel growth is reassuring. An oblique line converging on the physis signals a bar
Distal tibia	III/IV (incl. Tillaux and triplane). Younger SH III/IV	Moderate–high. Age-dependent, low near maturity	Physeal or articular gap or step-off >2 mm	Every 3–6 months for 12–24 months (less if near maturity)	Asymmetric arrest produces progressive angulation
Distal radius	I/II (common). III/IV (uncommon)	Low (~1–7%), rising with repeated manipulation	Displaced SH III/IV. Irreducible SH I/II	6–12 months. Longer if a bar is suspected	A line paralleling the physis confirms resumed uniform growth
Proximal humerus	I/II	Low. Contributes ~80% of humeral length, so shortening is well tolerated	Rarely, open, neurovascular or irreducible injuries only	6–12 months	Rarely of clinical consequence given the low arrest rate

## Data Availability

No new data were created or analyzed in this study. Data sharing is not applicable to this article.

## Abbreviations

The following abbreviations are used in this manuscript.

ACL	anterior cruciate ligament
AIIS	anterior inferior iliac spine
ALARA	as low as reasonably achievable
ASIS	anterior superior iliac spine
AVN	avascular necrosis
BMI	body mass index
CT	computed tomography
IM	intramedullary
MRI	magnetic resonance imaging
MT	metatarsal
PLC	posterior ligamentous complex
SH	Salter–Harris
TLSO	thoracolumbosacral orthosis
